# Dual Perspectives on Peptide–Zinc Complexation: Highlighting Aquatic Sources While Contextualizing Other Natural Origins

**DOI:** 10.3390/biom15091311

**Published:** 2025-09-12

**Authors:** Lingyu Han, Nuo Dong, Jixin Yang, Bing Hu

**Affiliations:** 1 Key Lab of Biotechnology and Bioresources Utilization of Ministry of Education, College of Life Science, Dalian Minzu University, Dalian 116600, China; 20191398@dlnu.edu.cn (L.H.); 202312040020@dlnu.edu.cn (N.D.); 2 Faculty of Social and Life Sciences, Wrexham University, Plas Coch, Mold Road, Wrexham LL11 2AW, UK; j.yang@glyndwr.ac.uk

**Keywords:** zinc chelating peptides, zinc binding, zinc absorption, bioavailability, functional features

## Abstract

Zinc is an essential mineral for the body, with chelated zinc valued for its superior absorption efficiency and bioavailability. This review systematically examines peptide–zinc interactions, covering fundamental concepts, historical evolution, current insights, clinical relevance, technological innovations, and future outlooks. It delves into chelation mechanisms and structural theories, summarizes historical milestones in bioavailability research—particularly aquatic protein–zinc interactions—and details current studies on chelation efficacy and interaction dynamics. Clinical applications in nutritional supplements, therapeutic potential, and trial progress are discussed, alongside advances in analytical techniques, complex synthesis, and computational modeling. Future directions highlight emerging trends, application prospects, and challenges in bioavailability research, offering a comprehensive framework for subsequent investigations and practical implementations.

## 1. Introduction

**Figure 1:**
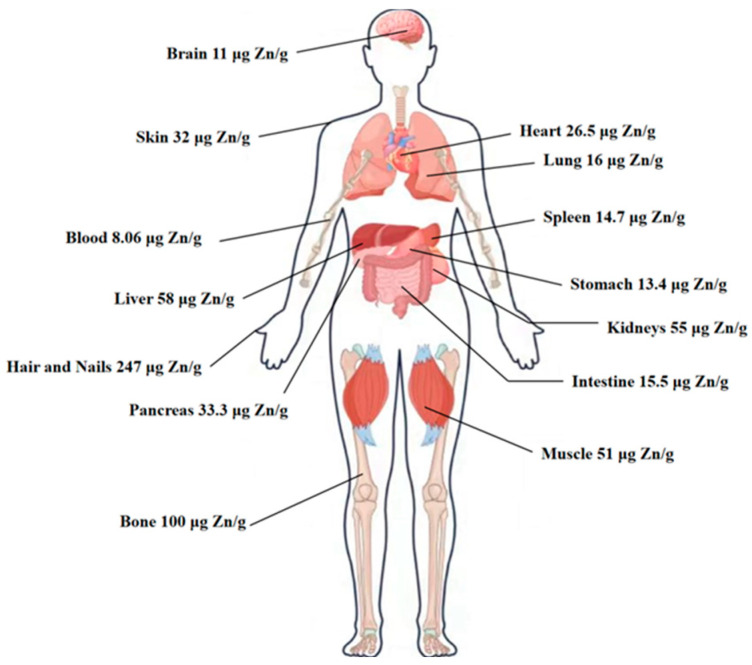
Overview of zinc distribution association in the human body. Approximate zinc content (µg per g wet weight) of the respective tissues and the resulting proportion of total body zinc [13].

The human body typically contains a total of 2 to 2.5 g of zinc as an essential nutrient, which is distributed across all tissues, organs, body fluids, and secretions, with relatively higher concentrations in the skin, bones, hair, prostate, gonads, and eyes (Figure 1). Zinc is the second most abundant trace element in the body after iron, and it is just as vital. Although the body requires only small amounts of zinc, the majority of it exists as a crucial component of numerous enzymes [1], which act as catalysts for many biochemical reactions. Without these enzymes, essential metabolic processes would slow down and eventually cease, highlighting zinc’s critical role in sustaining life [2]. Unlike iron, zinc cannot be stored in fat tissue, and there is no dedicated storage mechanism for it in the body.

Zinc levels in the human body are regulated by tissues including muscle, bone, and skin, with dietary intake being the primary source of supplementation. Various factors, such as the chemical composition of food, along with an individual’s nutritional, metabolic, and physiological status, influence its distribution and bioavailability [3]. Humans predominantly obtain zinc in its ionic forms, particularly through zinc sulfate (ZnSO_4_), zinc gluconate (ZnG), and zinc-fortified yeast (ZnY) [4]. However, ZnSO_4_ poses a challenge owing to its distinctly metallic, bitter, and astringent taste, which requires flavor-masking techniques to improve the palatability. Moreover, ZnSO_4_ frequently triggers side effects, including nausea and vomiting, making its application less favorable. On the other hand, while ZnG is an effective source, its high cost and low zinc concentration present significant drawbacks [5]. These limitations severely hinder the absorption efficiency and bioavailability of zinc from dietary intake.

Currently, research on the side effects of long-term intake of peptide zinc complexes on the human body is still limited. However, existing evidence suggests that within the recommended dosage range (adult daily zinc intake ≤ 40 mg), its safety is significantly higher than that of inorganic zinc supplements (such as zinc sulfate). Potential risks are primarily associated with excessive intake, including disruption of the absorption balance of other minerals (such as copper and iron), gastrointestinal discomfort, and renal metabolic stress due to high zinc load. Peptide–zinc complexes derived from aquatic organisms exhibit higher bioavailability and targeting, enabling equivalent zinc supplementation effects to inorganic zinc supplements at lower intake levels. Their long-term safety is superior to that of terrestrial-sourced or chemically synthesized complexes; however, further clinical data are needed to support this conclusion.

The habitat characteristics of marine organisms (such as depth and salinity) are significantly associated with the sequence structure, coordination mechanism, and functional characteristics of their zinc-chelating peptides. Extreme environments (such as deep-sea high pressure, high-salinity waters, and polar low temperatures) have shaped the adaptive characteristics of zinc-chelating peptides within organisms through evolutionary pressure—for example, deep-sea organisms often enhance coordination stability to cope with low-zinc environments, while organisms in high-salinity regions have evolved unique structures resistant to ionic interference. This “habitat-peptide feature” association constitutes the crucial molecular basis for marine organisms’ adaptation to zinc ion homeostasis regulation and serves as a key evolutionary marker distinguishing aquatic-derived peptides from terrestrial peptides.

Chelated zinc emerges as a promising alternative to ionic zinc, addressing two key limitations as mentioned above. It is widely recognized for improving the bioavailability of dietary minerals, leading to increased absorption and more efficient consumption. Moreover, it significantly decreases the risk of mineral deficiencies, positioning it as a more advantageous option compared to conventional ionic forms [3]. Amino acid-chelated zinc and peptide–zinc complexes maintain solubility across varying pH levels during digestion, thereby retaining their electrically neutral state. The absorption efficiency of zinc in these chelates is significantly affected by the choice of ligand. Small peptides, in particular, provide several advantages over amino acids: they require less energy for absorption, facilitate rapid transport, and reduce the risk of carrier saturation, thereby enhancing zinc uptake [6].The primary functional groups responsible for coordinating Zn^2+^ in different natural peptides are directly related to their amino acid composition. Polar functional groups containing nitrogen and oxygen atoms are key coordination sites, specifically including the imidazole group (-C_3_H_3_N_2_ containing a coordinating nitrogen atom) of histidine (His), glutamic acid (Glu) and aspartic acid (Asp) carboxyl groups (-COOH, which form -COO^−^ after deprotonation and coordinate via oxygen atoms), tyrosine (Tyr) phenolic hydroxyl groups (-OH, where oxygen atoms can participate in weak coordination or hydrogen bond-assisted coordination), and cysteine (Cys) sulfhydryl groups (-SH, the sulfur atom participates in coordination under specific conditions, commonly found in peptides derived from anaerobic organisms). These functional groups exhibit unique combination patterns in aquatic-derived peptides (such as the synergistic effect of high ratios of His and Glu), which is the core reason for their superior zinc chelation capacity compared to most terrestrial peptides. Casein phosphopeptide (CPP), characterized by its distinctive sequence of three phosphoseryl residues followed by two glutamate residues (SpSpSpEE), demonstrates a potent chelating ability towards divalent metal ions including iron and zinc. This property allows CPP to effectively form chelates with zinc, rendering it an ideal addition to fortified foods for improved mineral bioavailability [7]. Hence, peptide-chelated zinc emerges as a promising supplement for enhancing zinc absorption efficiency in the human gastrointestinal tract.

Due to environmental factors, marine-derived peptides have developed their unique structures. Marine organisms (such as sea cucumbers, seaweed, and fish) have long adapted to extreme environments characterized by high salinity, high pressure, and low temperatures. As a result, their peptide segments often evolve sequences rich in acidic amino acids (Asp, Glu) and histidine (His). For example, the proportion of Asp residues in seaweed peptides can reach 15–20% (significantly higher than the 5–8% in terrestrial plant peptides), and their carboxyl groups (-COOH) can form stable coordination bonds with Zn^2+^; The imidazole group of histidine (pKa ≈ 6.0) is easily protonated at physiological pH, forming strong coordination with Zn^2+^ (with dissociation constants Kd as low as 10^−8^–10^−9^ M) [8] This is the core reason why marine peptides exhibit superior zinc chelation capacity compared to most terrestrial peptides. (Seaweed Proteins as a Source of Bioactive Peptides) Marine peptides not only have high chelation efficiency, but also feature low toxicity and high biocompatibility (e.g., the allergenicity of fish collagen peptides is only 1/5 that of terrestrial animal peptides) [9]. Additionally, certain peptides (such as spirulina peptides) can exhibit antioxidant and anti-inflammatory activities while chelating zinc, making them suitable for the composite requirements of functional foods and pharmaceutical applications [10]. Secondary structures (such as α-helices) can stabilize chelation sites through spatial arrangement. For example, the α-helix structure of Antarctic krill peptides maintains the distance between ligand residues (Glu, His) at 0.3–0.5 nm (matching the coordination radius of Zn^2+^), resulting in a half-life of 8 h for the chelate in simulated gastrointestinal fluid (only 2 h for peptides with random coiled-coil structures) [11]; the “hydrophobic pockets” formed by tertiary structures can encapsulate Zn^2+^, avoiding competitive binding with high concentrations of Ca^2+^ and Fe^3+^ in the gastrointestinal tract, thereby enhancing complex stability. Take oyster peptides as an example. In addition to chelating zinc through the “-Asp-Glu-” sequence (with a binding rate of 92%), they can also exert immune-modulating effects through sulfur-containing amino acids (such as cysteine) [12]. In infant zinc supplements, they can simultaneously reduce the risk of infection, which is an advantage that single-function peptides cannot replace.

This paper offers a thorough review of recent advancements in the research of zinc chelating peptides, covering marine-derived sources, the mechanisms of zinc–peptide chelation, and the role of these peptides towards enhanced zinc absorption and bioavailability.

## 2. Peptide–Zinc Chelation Model

**Figure 2:**
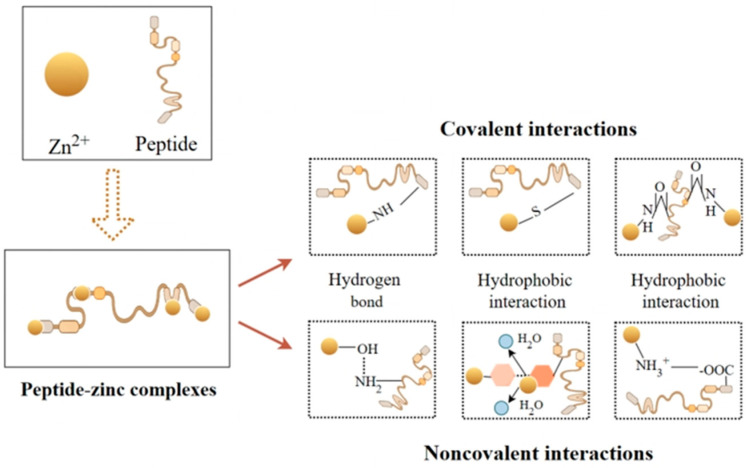
Interaction between protein peptides and zinc ions.

Understanding the complex interactions between zinc ions and protein peptides is essential for the strategic development of foods and ingredients with novel or improved functional properties (Figure 2). Studies have demonstrated that zinc preferentially binds to particular sites of peptides. Phosphate group and carboxyl group are the most frequently reported amino acid groups contributing to zinc chelating activity. This initial understanding guides the optimization of food formulations, enabling the development of innovative products that leverage these interactions for enhancing health benefits and performance [14]. Chelation of zinc by peptides involves specific chemical interactions between the peptide and zinc ions. Casein-derived peptides have shown promise in improving zinc bioavailability through chelation. For instance, the novel 11-residue peptide TEDELQDKIHP identified from casein hydrolysate chelates zinc through the carboxyl groups (from Asp and Glu), amino groups (from Lys and His), pyrrole nitrogen group of Pro, and imidazole nitrogen group of His [14]. The complexation with zinc results in a more ordered structure of TEDELQDKIHP-Zn. In terms of digestion stability, the chelate can remain stable to a large extent after gastric (78.54 ± 0.14%) and intestinal digestion (70.18 ± 0.17%).

Chickpea peptides also interact with zinc ions. The peptide HKERVQLHIIPTAVGK from chickpea glutelin hydrolysate shows a relatively high chelating capacity (57.86 ± 2.14%). Spectral methods prove that side-chain and C-termini carboxyl groups of the peptide mostly participate in chelation, and N atoms of amino side-chains, the imidazole group of histidine, and N-termini are also involved to some extent [15]. According to ICP-OS analysis, 1 mg peptide can chelate 381.61 ± 133.39 μg zinc, and the molar ratio of peptide–zinc is about 1:4.

### 2.1. Theoretical Models of Peptide–Zinc Interactions

Theoretical models can provide insights into the interaction of peptides with zinc. While there are no direct theoretical models of peptide–zinc interactions in the given references, studies on the interaction of glycine with hydrogenated amorphous carbon (HAC) at the Density Functional Theory (DFT) level offer a methodological reference [16]. This approach can potentially be applied to study peptide–zinc interactions, as it allows for the exploration of the forces and energies involved in the interaction.

In addition, models for peptide homodimerization can also provide some inspiration. By understanding how simple interaction models can explain sequence-dependent effects in peptide homodimerization, similar principles may be applied to peptide–zinc interactions. For example, the use of statistical methods to assess whether simple interaction models can capture complex phenomena can be extended to study peptide–zinc binding, helping to identify the key factors influencing their interaction.

### 2.2. Phosphate Group–Zinc Chelation Pattern

Casein phosphopeptides (CPP) are significant phospho-zinc chelating peptides characterized by their strong phosphorylated nature. Derived through enzymatic digestion of casein proteins, CPP includes phosphorylated segments from αs1-, αs2-, and β-casein. This peptide features a distinctive amino acid sequence of Ser(P)-Ser(P)-Ser(P)-Glu-Glu [7], incorporating three phosphorylated serine residues and two glutamic acid molecules. The findings of the study highlight the ability of CPP to bind essential bivalent cations, a property attributed to its acidic functional domain [17].

CPP enhances the bioavailability of zinc in zinc sulfate solutions in a dose-dependent manner [18]. At zinc-to-CPP ratios of 1:1 and 1:2, there was a statistically significant increase in bioavailable zinc at the 95% confidence level, while a ratio of 1:3 resulted in an even higher enhancement, reaching statistical significance at a 99% confidence level. This also confirms the findings of Lönnerdal et al. and Pérès et al. who suggested that zinc is effectively absorbed in aqueous solutions in the presence of CPP [19,20]. In the 1990s, research on CPPs demonstrated their role in enhancing zinc absorption, particularly in diets high in phytic acid, such as those containing oat foods. Zinc ions can form complexes with β-casein (1–25), resulting in a zinc absorption rate of 30.03% in rabbits, significantly surpassing the 20.05% absorption rate observed with ZnSO_4_ [14]. The weak bonding between zinc and phosphoserine residues in CPP plays a crucial role in its nutritional efficacy by allowing the gradual release of zinc and other minerals into the intestinal lumen, thereby enhancing their absorption [18].

### 2.3. Carboxyl–Zinc Chelation Method

Previous studies have identified various non-phosphorylated peptides capable of binding with zinc via carboxyl groups of amino acids. Liu et al. investigated zinc-binding peptides from sea cucumber hydrolysate (ZCPs) and found a direct correlation between their zinc-binding capacity and the carboxyl group content [21]. Aspartic acid (Asp) and glutamic acid (Glu) were identified as key carboxyl groups involved in zinc binding. Furthermore, the effectiveness of zinc-binding was influenced by the molecular weight of the peptides, with those under 1 kDa demonstrating a significantly higher affinity. The results indicated that after binding, zinc-chelated sea cucumber peptides might polymerize and form unique structural configurations, which may enhance their effectiveness in zinc absorption [22].

In addition to sea cucumber peptides, Sun et al. isolated three specific zinc-binding peptides from scallop adductase hydrolysates (SAH), namely VDAALAK, DLGDIK, and VTLEGK [4]. Ultimately, three major intestinal transport peptides were identified, each approximately 600 Da in size, from the serosal side of rat intestinal sacs. Synthetic versions of these peptides demonstrated increased zinc-chelating abilities and effectively traversed intestinal membranes. However, their permeability was slightly decreased, probably due to their charge and hydrophobic characteristics [23]. Hydrolyzed oyster protein with pepsin was purified to produce a novel zinc-binding peptide, HLRQEEKEEVTVGSLK. UV-vis and FTIR analyses identified the amino nitrogen and carboxylate oxygen atoms as the main sites for Zn^2+^ binding [24].

Despite identifying crucial groups for zinc chelation, further investigation is still required to understand the exact locations of chelating sites, molecular structures, and dynamic mechanisms along the peptide chain.

### 2.4. The Key Role of Aromatic Amino Acids in Peptide–Zinc Coordination

The cyclic structure and functional groups of aromatic amino acids (histidine, tyrosine, tryptophan) are important sites for peptide coordination with zinc ions (Zn^2+^). Their mechanism of action is closely related to charge characteristics and spatial configuration, and is particularly significant in peptides derived from aquatic sources [25].The imidazole ring of histidine (containing two nitrogen atoms) is the most common core site in peptide–zinc coordination. The lone pair of electrons on its nitrogen atom can form a stable coordination bond with Zn^2+^, and the pKa of the imidazole group (≈6.0) is close to physiological pH, enabling it to easily exist in a protonated and deprotonated equilibrium state in the body fluid environment, flexibly adapting to the coordination requirements of Zn^2+^ [26]. Although the phenolic hydroxyl group (-OH) of tyrosine is less reactive than the imidazole group, it can participate in Zn^2+^ coordination through oxygen atoms in specific sequences or stabilize the coordination environment through hydrogen bonds. The hydrophobic effect of its aromatic ring can also fix the peptide chain conformation through spatial stacking, preventing the coordination site from being exposed due to disordered folding.

## 3. Production and Purification of Zinc Chelate Peptides from Proteins of Marine Origin

**Figure 3:**
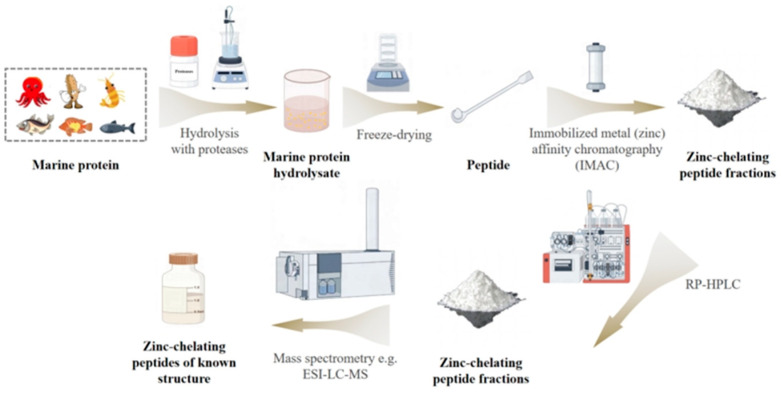
Bioassay of zinc chelate peptides in marine protein hydrolysates to facilitate purification and identification based on Udechukwu et al. Methods [3].

The production and refinement of zinc chelate peptides derived from food-grade proteins generally follow a well-established bioassay-guided approach (Figure 3). The process is initiated with the strategic selection of a protein known for its ability to bind zinc or other divalent metals. This approach not only optimizes the use of valuable proteins but also adds economic value to underutilized food sources or by-products, especially those derived from marine environments.

The screening of zinc chelating activity in peptides should take into account both “known-guided” and “discovery-guided” approaches: On one hand, peptides can be obtained from proteins known to have zinc-binding capabilities (such as histidine-rich metallothioneins and lactoferrins containing acidic amino acid clusters). Targeting the hydrolysis of their active regions (e.g., the ‘His-Asp’ motif in lactoferrin) can enhance screening efficiency, which is a ‘targeted mining’ strategy based on existing knowledge. After identifying the target protein source, it experiences the process of hydrolysis to convert it into a form suitable for further processing with specific proteases. Post-hydrolysis, the selective enrichment of zinc-chelating peptides is accomplished through advanced chromatographic purification strategies, wherein IMAC plays a pivotal role by leveraging metal-ligand interactions for targeted peptide recovery. IMAC exploits the inherent affinity of proteins to metal ions that are immobilized on the column matrix. This interaction forms stable but reversible complexes. By adjusting elution conditions, such as ionic strength and pH, the target peptides can be selectively released from these complexes.

On the other hand, the discovery of novel zinc-chelating peptides relies more on exploring unknown sequences, such as through the following mechanisms. Perform total component hydrolysis on proteins from biological resources with unreported zinc-chelating activity (e.g., extremophile microorganisms, lower aquatic organisms), combined with ICP-MS or fluorescent probe methods to screen for peptide components with zinc-binding capabilities, followed by mass spectrometry to identify their sequences (e.g., the novel hexapeptide ‘Glu-Met-His-Ala-Glu-Lys’ discovered in myosin from deep-sea krill, whose original source protein had no known zinc-chelating function).

Particularly, IMAC offers high sample recovery rates due to its gentle elution protocols involving non-denaturing solvents, ensuring minimal loss of bioactive components. When higher purity is required, peptides isolated through IMAC can be further purified using techniques such as size exclusion chromatography or reverse-phase high-performance liquid chromatography.

Following purification, the precise sequence of the zinc chelate peptides is determined using advanced mass spectrometry techniques, including MALDI-TOF/MS and LC/MS/MS. This crucial step identifies the peptides and reveals the amino acid residues responsible for their zinc-chelating properties, thereby providing valuable insights into their structure-function relationships and potential applications.

## 4. Zinc-Chelating Peptides of Marine Origin

The enzymatic method is widely used for the production of bioactive peptides. A previous study has extensively reviewed the applications of various proteases for protein hydrolysis [27]. Proteases play an essential role in the hydrolysis process, producing a wide array of hydrolytic products with varying molecular weights and distinct biological activities, including antioxidant properties [28], angiotensin-I-converting enzyme (ACE) inhibiting activity [29], immunomodulatory [30], and mineral binding [31]. The selection of protease is guided by the specific characteristics desired in the bioactive peptide. Various proteases have been reported to reproduce zinc-binding peptides, such as pepsin [32], trypsin [33], papain [34], protease M and flavor enzymes [35]. The selection of proteolytic enzymes is based on their ability to cleave specific peptide bonds, particularly those involving the carbonyl carbon that can be targeted by the catalytic group’s nucleophilic attack.

The crystal structure of peptide-bound neprilysin, a zinc metalloprotease, reveals key binding interactions [36]. The structure provides evidence for a potential exosite within the central cavity that may play a critical role in substrate positioning. Understanding these structural features is crucial as they can influence the function of the peptide–zinc complex and its biological activity.

Currently, zinc chelating peptides have been successfully isolated and characterized from marine sources, particularly from proteins and their hydrolysates of oyster [37], alaska pollock skin [38], marine octopus [39], antarctic krill [4], sea cucumber [21,23], pufferfish skin [40], tilapia scales [41], and tilapia skin [42], scallop [43] and scallop adductor [44], and silver carp [45]. Based on the data from Supporting Table S1, Figure 2 displays the zinc chelating peptides identified to date from various marine sources.

Research on the interaction between peptides of aquatic origin and zinc has yielded some key findings. For example, auxotrophic interactions in aquatic microbial communities have been explored, and it has been proposed that these interactions may influence the stability of these communities [46]. Although not directly about peptide–zinc interactions, understanding the complex web of metabolic dependencies in aquatic environments can provide context for how proteins in these environments may interact with zinc.

The biophysicochemical interactions at the interfaces between nanoparticles and aquatic organisms, including adsorption and internalization, have also been studied [47]. These findings can be related to how various forms of zinc present in aquatic environments interact with aquatic peptides. Environmental parameters such as pH fluctuations, ionic strength, and natural organic matter content could further modulate protein–zinc interfacial interactions in aquatic environments.

**Figure 4:**
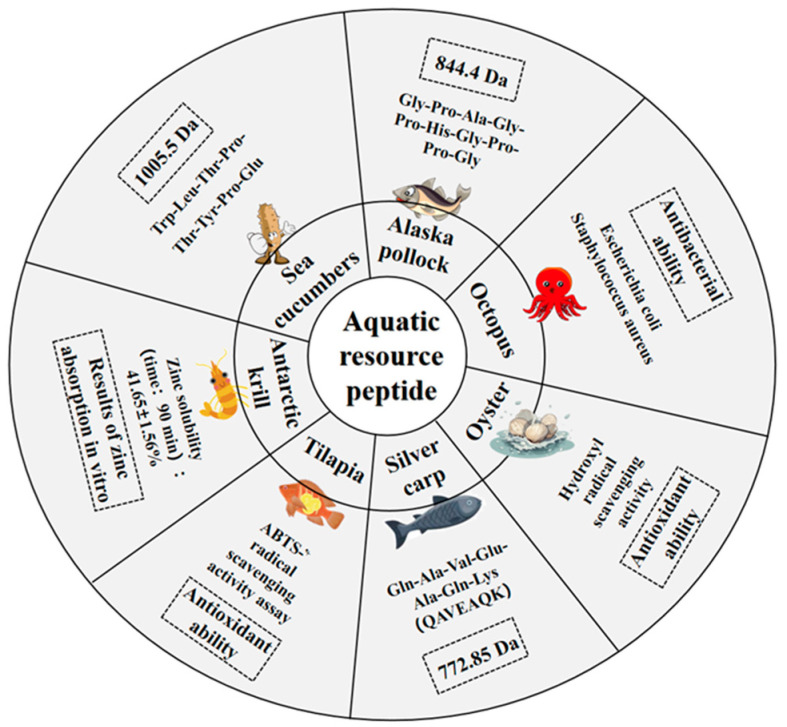
Peptides extracted from various marine sources, demonstrating diverse biological properties, including antioxidant and antimicrobial activities, molecular weight, and zinc retention following intestinal transport. The data source is Supporting Table S1.

Although there is a lack of extensive research directly on the structural analysis of peptide–zinc complexes in the provided references, studies on other peptide-protein complexes can offer insights. For example, the structural analysis of peptide-HLA complexes help in understanding how peptides interact with proteins, which may share some similarities with peptide–zinc interactions in terms of the forces and geometries involved in complex formation. These structural details can guide the design of peptides with enhanced zinc-binding capabilities and better understanding of their biological functions (Figure 4).

### 4.1. Aquatic Sources

Aquatic organisms, contributing to approximately half of global biodiversity, contain a rich array of structurally diverse bioactive compounds with a wide range of biological activities [48]. Protein-rich aquatic organisms are valuable sources of bioactive peptides for food applications. Furthermore, various bioactive peptides have been extracted from by-products of aquatic product processing, which are commonly discarded or repurposed as animal feed or fertilizer [49]. These peptides demonstrate promising biological functionalities and have led to various proposals for functional food applications [48]. Over the past decade, a variety of zinc-chelating peptides have been identified in aquatic products and their by-products, highlighting their potential significance.

#### 4.1.1. Tilapia

Tilapia, a major freshwater fish species, has been identified as one of the most farmed fish worldwide, alongside carp, catfish and salmon. Modern taxonomy classifies Nile tilapia as belonging to the genus Oreochromis, which includes approximately 30 species, characterized by maternal mouthbrooding behavior and euryhalinity. It is a nutritious choice for a balanced diet, offering high protein content (ranging from 16% to 25%) and low-fat levels (over a range of 0.5% to 3.0%). Tilapia serves as a versatile alternative in various seafood recipes [41]. The zinc chelation capacity of peptides, their solubility at different molecular weights, and the stability of the formed complexes have been assessed through simulated in vitro digestion studies [42,50]. It was concluded that peptides with lower molecular weights demonstrated improved zinc chelation ability, probably due to the presence of a diverse range of metal-binding amino acid residues. Meng et al. utilized collagen peptide hydrolysates derived from tilapia fish scales to prepare zinc nanoparticles chelated with collagen peptides [42]. Their findings that among hydrolysates treated with ten different enzymes, trypsin-derived hydrolysates exhibited the most effective nano-chelating capability. Meng et al. studied the properties of zinc chelates derived from collagen peptides in tilapia fish skin, using zinc sulfate (P-Zn-S) and zinc lactate (P-Zn-L) as starting materials [42]. Their findings indicated that P-Zn-S demonstrated a higher zinc chelating ability and a distinct structural morphology, potentially attributable to its amino acid profile, particularly Asp, Glu, His, Lys, Arg, Cys, and Pro. In summary, the peptides prepared by tryptic hydrolysis of tilapia with low molecular weight, chelate zinc ions in zinc phosphate, have good zinc chelation ability and maintain stability over in vitro simulation of gastrointestinal digestion.

#### 4.1.2. Oyster

Oysters are identified as the most widely cultivated bivalves worldwide, representing a crucial marine resource for human use and one of the most commonly consumed shellfish in China’s coastal regions. In traditional taxonomy, the genera Ostrea and Crassostrea exhibit paraphyletic issues. The branch containing Pacific oysters forms an independent monophyletic group, having diverged from other species in the Crassostrea genus over 25 million years ago. International authoritative databases (such as GBIF (https://doi.org/10.1007/s10531-022-02458-x, accessed on 9 September 2025) and WoRMS (https://doi.org/10.1371/journal.pone.0051629, access on 9 September 2025)) have reclassified them into the Magallana genus to reflect their unique evolutionary history. Oysters have significant economic value, with their soft tissues being edible and highly nutritious. Furthermore, oyster shells serve as raw materials in traditional Chinese medicine and lime production, and as feed for poultry. Hydrophilic and hydrophobic polypeptides produced via oyster protease-mediated hydrolysis have been reported to exhibit distinct zinc-binding behaviors, where hydrophobic peptides engage in zinc complexation through carbonyl covalent bonds and amino group coordination. Furthermore, Nano LC-Q-TOF analysis identified aspartic acid, glutamic acid, and leucine as key amino acids potentially influencing the zinc chelation capacity [37]. In another study, the synthesis of an oyster protein hydrolysate-zinc (OPH-Zn) complex was investigated for its impact on zinc bioavailability [51]. It revealed that carboxyl, carbonyl, and amino groups, associated with various charged amino acids, serve as primary binding sites for zinc. Furthermore, the OPH-Zn complex retained or even enhanced its antioxidant properties after digestion, suggesting its potential as a functional ingredient that improves both antioxidant activity and zinc bioaccessibility. Meanwhile, pepsin was used to hydrolyze oyster protein and isolate a distinct zinc-binding peptide [24]. Sequence analysis with LC/LTQ identified the peptide as His-Leu-Arg-Gln-Glu-Glu-Lys-Glu-Glu-Val-Thr-Val-Gly-Ser-Leu-Lys, revealing a specific sequence of amino acid from N- to C-terminus. In summary, oyster peptide-chelated zinc maintained not only good stability over in vitro gastrointestinal simulation digestion, but also its antioxidant properties. In addition, the unique sequence of oyster peptides prepared by pepsin enzymolysis can be isolated for further study.

#### 4.1.3. Patinopecten Yessoensis

Patinopecten yessoensis, an extensively cultivated shellfish in East Asia, produces scallop cladding as a by-product during industrial processing. Although rich in protein and valuable as a protein source, scallop cladding is frequently disregarded by numerous food processing facilities. Studies have been conducted using this product as a processing by-product. The influence of scallop mantle hydrolysates (SMHs) on zinc absorption in the intestines has been explored. The research focused on the degree of hydrolysis (DH) and protein solubility of the hydrolyzed products, demonstrating that alkaline protease is particularly effective for extracting peptides from scallop mantle proteins. The molecular weight distribution of the resulting SMHs primarily ranges from 1 to 5 k Da. Importantly, zinc chelation with SMHs, involving both carboxyl and amine groups, does not significantly alter its secondary structural conformation. Furthermore, several representative transport peptides, in both intact and fragmented forms, were identified, including FTGEPGPSGPT, AINDPFIDL, and ALPHAILRI [43]. Sun et al. prepared a food-derived zinc carrier using scallop shell harvest enzyme hydrolysate (SAHs) as a carrier [44]. The study found that while the Zn-chelating ability of SAH decreased during temporary storage at 4 °C, the SAH-Zn complex remained highly stable. The secondary structure of SAH remained largely unaffected. However, Zn influenced the surface structure of SAH, primarily affecting its carboxyl groups. Digestion in the gastrointestinal tract demonstrated the strong stability of the SAH-Zn complex, with minimal degradation of its high molecular weight components. Furthermore, three synthetic peptides demonstrated improved zinc chelating ability compared to SAH, while also effectively traversing the intestinal barrier [44]. In summary, several representative peptides can be isolated from patinopecten yessoensis peptide prepared by alkaline protease hydrolysis. In addition, the patinopecten yessoensis peptide zinc chelate maintained good stability during storage and in vitro gastrointestinal mock digestion.

#### 4.1.4. Alaska Pollock

The Alaska pollock is a commercially significant fish, with approximately 500,000 tons processed each year. Its skin and backbone, by-products of aquatic product processing, are frequently discarded or employed to produce low-value feed. The researchers identified the amino acid sequence Gly-Pro-Ala-Gly-Pro-His-Gly-Pro-Pro-Gly (GPAGPHGPPG) from zinc-chelating peptides derived from the skin of Alaska pollock. Chen et al. found that approximately 75% of GPAGPHGPPG remained intact after in vitro gastrointestinal enzyme digestion [38]. The coordination of this peptide segment with zinc ions may be attributed to the dehydrated state of histidine and its chelating ability due to its heterocyclic structure. Mineral ions can be transported across cell membranes in the form of chelates, significantly enhancing the transport efficiency of zinc ions in Caco-2 cells [38]. In summary, the Alaska codfish peptide zinc chelate not only exhibits good stability in in vitro gastrointestinal simulated digestion but also effectively promotes the absorption and transport of zinc ions by Caco-2 cells, making it a potential nutritional component for functional foods aimed at preventing mineral deficiencies.

#### 4.1.5. Antarctic Krill

Antarctic krill (Euphausia superba), an abundant crustacean in the Antarctic Ocean with biomass potentially exceeding 1 billion tons, represents a promising marine resource [52]. Sun et al. developed a novel peptide–zinc chelate—AKP-zinc [4]—from Antarctic krill peptides (AKP). The reaction was conducted at pH 6.0 and 60 °C, with a mass ratio of AKP to ZnSO_4_·7H_2_O of 1:2 and a reaction time of 10 min. The study found that zinc can form stable complexes with the carboxyl and amino groups of AKP. These AKP-zinc chelates exhibit higher stability across a range of pH values and during simulated gastrointestinal digestion, outperforming zinc sulfate and zinc gluconate in terms of performance.

#### 4.1.6. Octopus

Marine octopus, a plentiful resource in Asia, is highly valued for its rich nutritional profile [53]. With increasing global demand, the processing of octopus led to substantial by-products, including viscera, eyes, and other wastes, which present significant environmental challenges [54]. The researchers used these waste proteins to form new zinc-chelating peptides that have antibacterial activity against Escherichia coli and Staphylococcus aureus [39,55]. The peptide’s carboxyl group and amino nitrogen were identified as key zinc-binding sites. Mechanistic studies demonstrated that this zinc chelate disrupts bacterial membranes and walls while increasing permeability and causing cellular damage. These effects were evidenced by alterations in bacterial morphology, perturbations in membrane potential, accumulation of intracellular ROS, and modulation of enzymatic activities [39].

#### 4.1.7. Sea Cucumbers

In Asian cultures, sea cucumbers are highly valued as a tonic, renowned for their perceived health benefits. A study by Bordbar et al. has demonstrated that they offer a wide range of biological and pharmacological activities that contribute to overall health [56]. Sea cucumber body wall protein contains approximately 70% insoluble collagen fibers [57], which play a crucial role in maintaining the structural integrity of connective tissue’s extracellular matrix and are recognized as a rich source of numerous bioactive peptides [58]. Wang et al. synthesized a sea cucumber peptide–zinc chelate and characterized its digestive stability and absorption potential using in vitro models [23]. Isothermal titration calorimetry revealed that the zinc–peptide binding process was exothermic with moderate affinity. As shown by SEM, zinc chelation induced structural rearrangement of the peptide, resulting in irregular structures such as flakes and fragments in the sea cucumber peptide, while the sea cucumber peptide–zinc complex formed larger clusters with a reduced number of small clusters. At the same time, digestive stability was maintained. The complex demonstrated dual absorption pathways: paracellular transport in erosion rat intestine and transcellular uptake across Caco-2 epithelia. Liu et al. isolated and characterized a zinc-chelating peptide (ZCP) from sea cucumber body wall proteins following alkaline protease hydrolysis [39]. Isothermal titration analysis revealed an endothermic zinc-ZCP binding reaction with modest affinity. Fourier transform infrared spectroscopy (FTIR) analysis identified carboxylic acid and amide groups as the primary zinc-binding sites in ZCP. Ultra-high performance liquid chromatography coupled with Quadrupole/time-of-flight mass spectrometry (UPLC-Q-TOF-MS/MS) determined the representative ZCP sequence to be WLTPTYPE, with a molecular weight of 1005.5 Da [59]. In summary, sea cucumber peptide can not only isolate a representative sequence, but also maintain a good stability of zinc chelate of sea cucumber peptide during in vitro simulation of gastrointestinal digestion, intestinal sac and Caco-2 cell experiments in rats.

#### 4.1.8. Silver Carp

The silver carp (hypoothmichthys molitrix) is one of the most widely farmed fish in the world because of its rapid growth, ease of farming, and high nutritional value [60]. In China, silver carp are typically sold fresh, with the meat often shredded for producing frozen fish balls. Silver carp serves as a valuable protein source, enriched with essential amino acids such as Glu, Asp, His, Leu, Val, Ala, Thr, Ser, Gly, Ile, Tyr, Lys, and Arg [61]. However, its economic potential is severely hindered by a muddy flavor and high bone content, limiting its direct consumption. Therefore, it is primarily used in animal feed, resulting in considerable protein waste. Jiang et al. evaluated the antimicrobial properties of peptide–zinc complexes (PZCs) derived from silver carp protein hydrolysates using four different enzymes: papain, alkaline protease, flavor enzyme, and trypsin [45]. Among these, the PZCs obtained from flavor enzyme hydrolysates demonstrated the highest antibacterial activity. Chromatographic techniques were used to isolate peptides from flavor enzyme hydrolysates and eleven peptides were characterized using nanoflow liquid chromatography coupled with electrospray ionization mass spectrometry. Among these, five peptides including EDLAKALAKK, GKKTAEIEK, QAVEAQK, KELEEK, and YEESQAELEGSLK, were selected for synthesis. The results revealed that peptides with a higher proportion of acidic amino acids demonstrated greater zinc ion binding capacity, leading to PZCs with enhanced antibacterial activity compared to those with fewer acidic residues. Native silver carp protein is especially rich in acidic amino acids, specifically Glu and Asp, which account for about 39% of its total amino acid content. This high concentration of acidic residues contributes to the established antibacterial activity of the PZCs derived from silver carp hydrolysate.

## 5. Current Insights into Peptide–Zinc Bioavailability

### 5.1. Role of Aquatic Proteins in Zinc Bioavailability

Aquatic proteins can play a significant role in zinc bioavailability. Collagen peptides from pufferfish skin, when chelated with zinc to form CP-Zn nanoparticles, have shown to be more effective in improving zinc bioavailability in zinc-deficient rats compared to zinc gluconate and zinc sulfate [40]. The spherical, nanosized CP-Zn particles (~100 nm) exhibited excellent pH stability, attributable to their surface charge characteristics as measured by zeta potential. Structural elucidation demonstrated that zinc ions formed coordinate covalent bonds with both the amino terminus (NH_2_ → Zn) and carboxyl groups (COO^−^ → Zn) of collagen peptides, accounting for the observed stability. Caseinophosphopeptides can also enhance zinc bioavailability. They overcome calcium phytate inhibition on zinc bioavailability by retaining zinc from coprecipitation as zinc/calcium phytate nanocomplexes [62]. Under physiologically relevant conditions, caseinophosphopeptides dose-dependently retained zinc in solution against calcium phytate coprecipitation. The formation of single-crystal zinc/calcium phytate nanocomplexes (Zn/CaPA-NCs) with a size and ζ-potential of 10–30 nm and −25 mV, respectively, was mediated by caseinophosphopeptides. These nanocomplexes were found to deliver bioavailable nanoparticulate zinc in mouse jejunal loop ex vivo model and polarized Caco-2 cells.

### 5.2. Recent Advances in Peptide–Zinc Interaction Studies

Recent advances in peptide–zinc interaction studies have spanned multiple interdisciplinary fields, with insights from seemingly unrelated research areas offering valuable perspectives for understanding the complex mechanisms underlying these interactions. In the realm of food science, for instance, studies on the interactions between myofibrillar proteins (predominantly from muscle tissues of aquatic and terrestrial organisms) and volatile flavor substances have shed light on general principles of biomolecular binding that can be extrapolated to peptide–zinc systems [63]. Myofibrillar proteins, characterized by their intricate three-dimensional structures with exposed hydrophobic pockets and polar residues, interact with flavor compounds through a combination of physical binding (e.g., hydrophobic interactions, van der Waals forces) and chemical adsorption (e.g., hydrogen bonding, electrostatic attraction). These dual-mode interactions share striking similarities with the proposed mechanisms of peptide–zinc coordination, where peptides—especially those from aquatic sources, which often evolve to withstand dynamic marine environments—may utilize both non-covalent forces (such as hydrophobic encapsulation of zinc ions within folded domains) and specific chemical bonding (via amino acid side chains like histidine’s imidazole group or glutamic acid’s carboxyl group).

Furthermore, the regulatory effects of endogenous and exogenous factors on myofibrillar protein-flavor interactions provide a useful framework for exploring how environmental variables might modulate peptide–zinc binding. For example, changes in pH, ionic strength, or temperature can alter the conformational flexibility of myofibrillar proteins, thereby enhancing or weakening their affinity for flavor substances. This parallels the scenario in peptide–zinc systems, where aquatic peptides (adapted to fluctuating marine conditions, such as varying salinity or pH levels) may exhibit unique responsiveness to environmental cues. For instance, a peptide derived from marine fish muscle might undergo a pH-induced conformational shift that exposes hidden zinc-binding motifs, a phenomenon analogous to how myofibrillar proteins from cold-water fish adjust their structure to retain flavor compounds in low-temperature environments. Such cross-referencing highlights the value of leveraging insights from related biomolecular interaction studies to refine hypotheses about peptide–zinc behavior, particularly in the context of aquatic peptides with distinct evolutionary adaptations.

Molecular dynamics (MD) simulations have proven to be reliable in predicting the stability of peptide–zinc complexes; however, their predictive results are subject to limitations such as the accuracy of force field parameters, simulation duration, and the degree of system simplification. Therefore, these results should be validated in conjunction with experimental data. In parallel, technological advancements in biophysical techniques have expanded the toolkit for investigating peptide–zinc interactions, with Forster Resonance Energy Transfer (FRET) emerging as a promising method [64]. FRET, which relies on the non-radiative transfer of energy between a fluorescent donor and an acceptor molecule in close proximity (typically within 1–10 nm), has been widely used to probe protein–protein interactions, conformational dynamics, and ligand binding events. Its application to peptide–zinc systems holds significant potential for real-time, high-resolution monitoring of complex formation. By labeling peptides with a FRET donor (e.g., fluorescein) and zinc ions with a compatible acceptor (e.g., a metal-sensitive fluorophore or quantum dot), researchers can track the energy transfer efficiency as zinc binds to the peptide, providing quantitative data on binding kinetics and affinity. Moreover, FRET can capture subtle conformational changes in peptides upon zinc coordination—such as the transition from a random coil to an α-helical structure, a common feature in many aquatic peptides that enhances their metal-binding stability. This capability is particularly valuable for studying aquatic peptides, which often possess flexible backbones that allow for adaptive structural rearrangements to optimize zinc chelation. Additionally, FRET-based assays can be adapted to study the dynamics of peptide–zinc interactions under physiologically relevant conditions (e.g., in simulated gastrointestinal fluids or marine-like buffers), offering insights into how these complexes behave in their native or application environments.

Together, these cross-disciplinary insights and technological advancements underscore the multifaceted nature of peptide–zinc interaction research, while emphasizing the need to contextualize such advances within the unique properties of aquatic peptides—organisms that have evolved sophisticated mechanisms to harness and regulate metal ions in their challenging habitats.

## 6. Clinical Implications of Peptide–Zinc Interactions

### 6.1. Peptide–Zinc Complexes in Nutritional Supplements

Peptide–zinc complexes show significant promise as bioactive ingredients in nutraceutical formulations. Food-derived zinc-chelating peptides demonstrate enhanced mineral absorption kinetics and improved bioavailability, addressing critical public health concerns as zinc deficiency is associated with growth retardation and compromised immune competence [3]. Certain amino acid residues-particularly cysteine, histidine, serine, aspartate, and glutamate-demonstrate zinc-chelating capacity through their functional groups, forming water-soluble coordination complexes with the metal ion via donor atom interactions.

The stability of the peptide–zinc complexes to gastrointestinal digestion is critical. If the complexes can resist digestion, they are more likely to deliver zinc effectively to the body. This makes peptide–zinc complexes promising ingredients for nutritional supplements to address zinc deficiency.

### 6.2. Therapeutic Potential of Peptide–Zinc Chelation

Peptide–zinc chelation exhibits significant therapeutic potential in various diseases, particularly in Alzheimer’s disease research. A notable example lies in histidine-rich branched peptides, which have been specifically designed as high-affinity Zn (II) chelators, with aquatic-derived sequences showing unique advantages [65]. These peptides, some of which are inspired by zinc-binding motifs in marine organisms (such as the His-Glu-rich segments in shellfish proteins), can stably complex Zn (II) at physiological pH 7.4 and possess the capacity to bind two equivalents of Zn (II) ions through multidentate coordination.

Crucially, their role in Alzheimer’s disease prevention is directly linked to zinc chelation: excess free Zn (II) in the brain is a key driver of Aβ (1–40) aggregation, a process central to amyloid plaque formation. Studies have demonstrated that these histidine-rich peptides, especially those derived from aquatic sources with optimized His spacing (e.g., the repeating “His-X-His” motif in salmon skin peptides), can effectively sequester free Zn (II) and reduce its availability for promoting Aβ fibrillization. Specifically, aquatic-derived branched peptides have been shown to inhibit Zn (II)-induced Aβ (1–40) aggregation by up to 58% in vitro, a efficacy that surpasses many terrestrial-derived counterparts due to their unique conformational flexibility in complexing Zn (II) [65].In noise-induced hearing loss, disruption of zinc signaling, either via ZnT_3_ deletion or pharmacological zinc chelation, mitigated hearing loss in mice [66]. This indicates that zinc chelation can be a potential treatment strategy. The ability of peptide–zinc chelation to modulate zinc levels and signaling pathways makes it a promising approach for treating diseases related to zinc dysregulation.

### 6.3. Clinical Trials on Peptide–Zinc Bioavailability

Although there is a lack of direct clinical trial data on the bioavailability of peptide–zinc complexes in the existing literature, research on the chelation of metals (especially transition metals) with peptides/proteins can provide targeted references for the design of clinical trials. Such research shares a common scientific basis with the peptide–zinc system in terms of coordination mechanisms and absorption barriers (such as stability and transport pathways).

For example, iron-binding peptides derived from aquatic sources (such as salmon hemoglobin hydrolyzed peptides) and zinc-binding peptides share structural similarities: both rely on the coordination of residues such as histidine and glutamic acid to form stable complexes, and both face similar challenges in the gastrointestinal tract—such as complex dissociation due to pH fluctuations and competitive binding with other metal ions in the diet. Preclinical studies on salmon iron peptides indicate that the bioavailability of their complexes depends on two key strategies: first, optimizing peptide chain length (200–1000 Da) to enhance the recognition efficiency of intestinal transport proteins (such as PEPT1); second, utilizing the self-assembly properties of peptides to form nanoparticles, thereby reducing the destruction of coordination bonds by gastric acid [67]. These optimization strategies targeting the stability and absorption pathways of metal-peptide complexes can directly guide the design of clinical trials for peptide–zinc complexes (e.g., selecting aquatic zinc peptides with similar molecular weight ranges, monitoring the impact of gastrointestinal pH on complex states).

Another important reference comes from studies on copper-metallothionein (an endogenous polypeptide rich in cysteine). Studies on the bioavailability of this complex indicate that the strength of the coordination bond between the metal and the peptide (dissociation constant Kd) must be controlled within the range of 10^−6^–10^−8^ M: if too strong, the metal cannot be released within cells; if too weak, it is prone to dissociation before absorption [68]. This principle directly informs zinc–peptide complex research—when designing clinical trials, it is essential to prioritize screening for aquatic zinc peptides with Kd values within the physiologically effective range (e.g., oyster peptide with Kd ≈ 5 × 10^−7^ M) and concurrently monitor the dynamic release efficiency of serum zinc, avoiding the sole pursuit of high chelation rates at the expense of bioavailability.

These specific studies on “metal-peptide chelation,” though focused on other metals such as iron and copper, share core mechanisms (balance between coordination bond strength and bioavailability, peptide structure regulation of intestinal absorption) highly relevant to the peptide–zinc system, providing scientific basis for clinical trial design (e.g., dose gradient settings, biomarker selection).

## 7. Technological Advances in Peptide–Zinc Research

### 7.1. Analytical Techniques for Studying Peptide–Zinc Interactions

Analytical techniques play a crucial role in studying peptide–zinc interactions. Electrospray ionization-mass spectrometry (ESI-MS) has been shown to be a powerful tool for studying peptide-metal interactions [69]. It can provide information on the stoichiometry of peptide-metal complexes, the specific amino acids to which the metal cations are bound, and the degree of association in these complexes. For example, it can determine the ratio of peptide to zinc in a peptide–zinc complex and identify the key binding sites on the peptide. Spectroscopic techniques, such as UV-Vis spectrophotometry, circular dichroism, and fluorescence spectroscopy, can also be used to study peptide–zinc interactions. UV-Vis spectrophotometry can monitor changes in the absorption spectrum of a peptide upon zinc binding, providing information on the complex formation. Circular dichroism can detect changes in the secondary structure of the peptide, which may occur due to zinc chelation. Fluorescence spectroscopy can be used to study the binding affinity and dynamics of peptide–zinc interactions.

### 7.2. Innovations in Peptide–Zinc Complex Synthesis

Innovations in peptide–zinc complex synthesis are emerging. The synthesis of a walnut peptides-zinc complex (WP1-Zn) has been reported, and this complex exhibits enhanced antiproliferative ability against human breast carcinoma cells through the induction of apoptosis [70]. The Zn ions were successfully combined with WP1 through Zn-N and Zn-O covalent bonds. This shows that novel peptide–zinc complexes can be synthesized with specific biological activities. Hierarchical reaction logic has enabled computational design of complex peptide syntheses [71]. This approach can be applied to the synthesis of peptide–zinc complexes, allowing for the planning of complete routes to complex peptide–zinc targets. It can incorporate protecting-group strategies and realistic pathway pricing, and can be performed in solid-state or solution modes, providing more efficient and targeted synthesis methods.

### 7.3. Computational Approaches to Peptide–Zinc Interaction Modeling

Computational approaches can help in modeling peptide–zinc interactions. The classical quantitative structure-affinity relationship (QSAR) approach can be used to statistically correlate structure features with binding affinities for protein-peptide complexes [72]. Although not directly applied to peptide–zinc interactions in the reference, similar principles can be extended. By deriving various structural descriptors from the peptide–zinc complex structure, such as physicochemical, geometrical, and constitutional parameters, and correlating them with binding affinities, we can gain insights into the factors influencing peptide–zinc binding. Computational approaches for modeling GPCR dimerization can also offer inspiration [73]. These approaches can be adapted to predict the dimerization or oligomerization of peptide–zinc complexes, which may be important for understanding their biological functions. Understanding the three-dimensional structure and interactions of peptide–zinc complexes through computational modeling can guide the design of more effective peptide–zinc chelates.

## 8. Effect of Peptides on Intestinal Zinc Absorption

Zinc absorption, a pivotal aspect of nutrition research, is influenced by both inherent food molecules and those produced during digestion. For example, phytates form insoluble complexes with zinc that cannot be broken down due to the lack of phytase [13]. Instead, peptides can enhance it. All of these food factors seem to work by affecting zinc solubility and therefore the intestinal zinc absorption pathway.

### 8.1. Intestinal Absorption Pathway

All the zinc required by our bodies is obtained from the diet, and it is widely believed that zinc absorption occurs through two different pathways: one involves the absorption of zinc as free ions, while the other involves zinc absorbed as complexes with specific amino acids [74].

Zinc absorption occurs across the entire small intestine in humans [75,76]. However, the primary site of absorption remains unclear. Research in rats indicated high absorption rates in the duodenum, ileum [77,78], or only in the ileum [79] or jejunum [75,80]. While in vivo data in humans is limited, small intestine perfusion studies in healthy individuals suggested that the duodenum and jejunum are key sites for zinc absorption. Specifically, these studies indicated the duodenum as the primary absorption site in the human small intestine [81] and jejunum [75].

The transcellular transport mechanism of peptide–zinc complexes differs significantly from that of free peptides, although there is some overlap between the two (e.g., dependence on the PEPT1 transporter). Due to their zinc ion content, these complexes alter charge distribution and spatial structure, thereby activating specific transport pathways not involved in free peptides (e.g., metal ion transporter synergism, intracellular de-zincation, and re-transport). This difference is particularly pronounced in peptides of aquatic origin: their complexes often enhance transmembrane efficiency through “transporter synergism” or “conformational adaptation” mechanisms, while free peptides primarily rely on the single PEPT1 pathway.

#### 8.1.1. Transcellular Pathway

**Figure 5:**
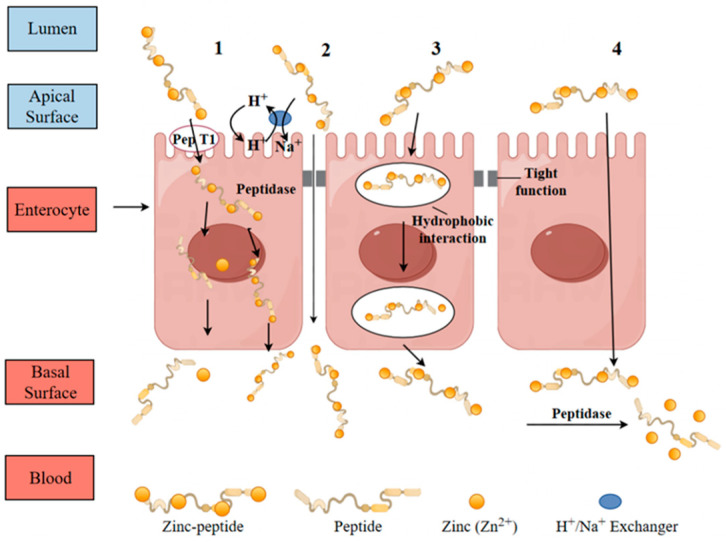
Peptide transport mechanisms including (1) carrier-mediated transport facilitated by pep T1, (2) paracellular permeation, (3) transcytosis, and (4) passive trans-cellular diffusion [22].

A proposed hypothesis suggests that bioactive peptides offer an alternative pathway for zinc transport through peptide-mediated mechanisms. After digestion, zinc bound to residual or newly formed peptides is thought to traverse the gastrointestinal epithelium and enter the bloodstream. The zinc–peptide complex may dissociate, allowing for the separate absorption of zinc and peptides, or it might be absorbed as a whole through mechanisms specific to peptide uptake. Currently, the precise mechanism by which zinc–peptide complexes are transported is not fully established. Figure 5 illustrates several proposed pathways, including carrier-mediated, paracellular, passive transcellular, and transendocytic transport mechanisms. These pathways are influenced by the structure of the peptides and the specific absorption sites in the epithelial cells [82].

#### 8.1.2. Paracellular Transport

Paracellular permeation is an energy-neutral process governed by the tight junction (TJ) architecture located between adjacent cellular interfaces. This TJ complex, comprising proteins such as zonula occludens-1, occludin, and claudins, forms a selective, semi-permeable barrier [83]. With an approximate pore size of 2.1 nanometers [84], it selectively allows the permeation of smaller, low molecular weight peptides. Examples of such peptides include Leu-Ser-Trp [85], Leu-Les-Pro, Ile-Gln-Trp [80], and Ile-Arg-Trp [86]. Particularly, paracellular transport provides an alternative pathway for larger peptides that cannot be absorbed through pepT1 without degradation. However, this pathway may involve partial enzymatic breakdown by proteases, leading to smaller peptide fragments [87]. This mechanism preferentially transports hydrophilic, negatively charged, short, and low molecular weight peptides [88].

The paracellular transport of peptide–zinc complexes (through the tight junctions between intestinal epithelial cells) depends on their specific structural characteristics: low molecular weight, moderate hydrophilicity, neutral net charge, and flexible peptide chains are key promoting factors. Peptides of aquatic origin, having evolved structures adapted to marine environments (such as shorter chains and balanced distribution of polar amino acids), exhibit significantly higher paracellular transport rates for their complexes compared to terrestrial peptides. The synergistic optimization of “flexibility-charge-size” is the core mechanism underlying this phenomenon.

### 8.2. Research Progress of Zinc Absorption Promoted by Peptides

Human intestinal cell models, particularly Caco-2 cells, are valuable tools for studying increased zinc absorption facilitated by zinc-chelated peptides in vitro [89].

Caco-2 cells have been widely used to study the absorption of iron and calcium ions in vitro [90]. Recent studies have explored the potential of CPP to enhance zinc absorption in the gut. Caco-2 cells, derived from human intestinal adenocarcinoma, demonstrated an intestinal epithelial-like characteristic when cultured on semi-permeable supports. This in vitro model closely resembles the native intestinal environment, making it suitable for evaluating the bioaccessibility of zinc in peptide–zinc complexes.

Chen et al. developed a chelating peptide, GPAGPHGPPG, derived from Alaska cod skin, which demonstrated improved zinc ion binding capacity [38]. This study assessed the stability of GPAGPHGPPG in simulated gastrointestinal enzyme digestion, examined potential binding interfaces between the peptide and metal ions, and evaluated its potential effect on mineral transport in Caco-2 cells. Approximately three-quarters (74.8%) of the GPAGPHGPPG peptide remained intact following simulated digestion. While zinc transport capacity enhanced by 32.3%, concomitant reductions in retention (60.2%) and absorption (13.8%) were observed. Structural analysis implies these phenomena may result from histidine-mediated chelation involving ring structure stabilization through dehydration.

## 9. Function and Application of Zinc Peptide Complex

**Figure 6:**
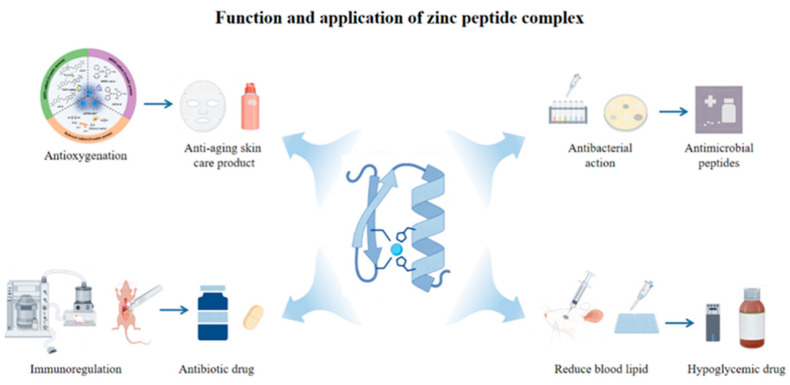
An illustration of the application of a peptide zinc complex.

The application of peptide–zinc complexes as functional components is increasingly recognized (Figure 6). This section examines their key characteristics and evaluates potential applications based on current research findings.

Zinc chelates formed with peptides can improve mineral absorption in the digestive tract by reducing the formation of insoluble zinc complexes and promoting the transport of zinc across the intestinal absorption barrier [91,92]. Sun et al. investigated zinc transport in its chelate hydrolysates derived from the adductor muscle of scallops (Patinopecten yessoensis) using an intestinal sac model in ectropion rats [44]. The study showed the successful transport of zinc across the intestinal barrier. Research has indicated that zinc chelates formed with oyster peptides can enhance antioxidant activity and improve zinc bioaccessibility [5]. More generally, these peptide-metal complexes are recognized for improving mineral bioavailability and displaying antimicrobial properties [93]. Lin et al. and Fang et al. showed that chelation of octopus scraps peptide with Zn^2+^ altered the peptide’s aggregation state [39,55]. The resulting chelated demonstrated significant antibacterial activity against both *E. coli* and *S. aureus*. This effect may be attributed to the disruption of cell membranes and the induction of oxidative stress-mediated apoptosis pathways. This study is the first to elucidate the synergistic effects of peptide and zinc complexes, highlighting their potential application as natural bacteriostatics in the food and pharmaceutical industries. The antibacterial activity of peptide–zinc complexes is driven by the combined effects of the characteristics of the peptide chain and the inherent antibacterial properties of zinc ions (Zn^2+^). Free Zn^2+^ itself exerts antibacterial effects through mechanisms such as disrupting bacterial cell membrane integrity (increasing permeability and causing the leakage of intracellular substances), inhibiting the activity of metal enzymes (such as iron-sulfur proteins in the bacterial respiratory chain), and interfering with DNA replication. Some aquatic peptides can recognize lipopolysaccharides (in Gram-negative bacteria) or teichoic acids (in Gram-positive bacteria) on the bacterial surface, directing the delivery of Zn^2+^ complexes to regions with high concentrations of pathogenic bacteria, thereby increasing the local effective concentration of Zn^2+^ by 3–5 times. Even if the peptide itself has no antibacterial activity, its Zn^2+^ complex still exhibits significant antibacterial effects. This is primarily attributed to the role of targeted Zn^2+^ delivery.

Due to rising concerns regarding chemical preservatives, the food industry is increasingly turning to natural GRAS (Generally Recognized As Safe) alternatives, emphasizing consumer preferences for safer additives [94]. Antimicrobial peptides (AMPs) have received significant attention as potential substitutes for chemical preservatives. These peptides, essential components of the innate immune system in various organisms, demonstrated increased antimicrobial activity when complexed with divalent metal ions such as zinc [95,96]. Studies revealed that peptide–zinc complexes (PZCs) derived from flavor enzyme hydrolysates demonstrated the highest antibacterial efficacy. Particularly, all five tested PZCs demonstrated significant antibacterial activity, although none displayed antifungal properties. Peptides with a higher content of acidic amino acids were found to bind more zinc ions, thereby enhancing the antibacterial activity of the PZCs [45].

The results showed that the zinc chelate also possessed antioxidant properties and promoted the proliferation of lactic acid bacteria. Furthermore, within the tested concentration range, higher levels of peptide–zinc chelate were positively correlated with scavenging of hydroxyl radicals, total reducing capability, and ABTS radical scavenging activity. Partial replacement of the organic nitrogen source in MRS medium with peptide–zinc chelate was found to promote the growth of lactic acid bacteria. However, when the organic nitrogen source was completely replaced with zinc chelate, both the acid production activity and the growth of human lactic acid bacteria were inhibited.

## 10. Conclusions and Future Trends

This review highlights the recent progress in the research of zinc-chelating peptides, focusing particularly on the ones of aquatic origins over the past few decades. CPP, known for its ability to bind zinc through a soluble complex formed by the phosphoric residue of serine, plays a crucial role in enhancing zinc absorption. Furthermore, non-phosphorylated peptides contribute to zinc absorption by forming carboxyl–zinc chelates with glutamate and aspartate residues, underscoring their significance in enhancing zinc bioavailability. Peptides can enhance zinc absorption by interacting with channels in the plasma membrane or by acting as carriers for zinc internalization into cells. Effective intestinal zinc absorption is essential for improving zinc bioavailability. Notably, zinc-binding or co-existing peptides tend to have a more significant impact on zinc absorption and bioavailability than non-zinc-containing peptides.

### 10.1. Emerging Trends in Peptide–Zinc Chelation Research

One emerging trend in peptide–zinc chelation research is the focus on peptides derived from natural sources. Casein-derived peptides, for example, have shown promise in improving zinc bioavailability through chelation [14]. Future research may further explore peptides from other natural sources, such as plant-based proteins, to identify novel peptides with enhanced zinc-chelating capabilities. These natural-derived peptides may have advantages in terms of biocompatibility and safety. Another trend is the study of the role of peptide–zinc chelation in disease prevention and treatment. As research progresses, more evidence is emerging on the potential of peptide–zinc chelation in diseases like Alzheimer’s and noise-induced hearing loss. Future research may expand these findings to other diseases related to zinc dysregulation, such as certain neurodegenerative and cardiovascular diseases.

### 10.2. Prospective Applications of Peptide–Zinc Complexes

Peptide–zinc complexes have prospective applications in various fields. In the area of drug delivery, peptide–zinc complexes can be designed to target specific cells or tissues. For example, by conjugating peptides with specific cell-targeting moieties, the peptide–zinc complexes can be directed to cells that are in need of zinc supplementation or where zinc-related signaling pathways need to be modulated. In the development of functional foods, peptide–zinc complexes can be used to enhance the nutritional value. Since zinc is an essential micronutrient, incorporating peptide–zinc complexes into foods can improve zinc bioavailability, addressing zinc deficiency in populations. The stability and bioactivity of peptide–zinc complexes make them suitable candidates for fortifying foods.

### 10.3. Challenges and Opportunities in Peptide–Zinc Bioavailability Research

One of the main challenges in peptide–zinc bioavailability research is the complexity of the gastrointestinal environment. The presence of various enzymes, pH changes, and other factors can affect the stability and absorption of peptide–zinc complexes 3. Understanding how these factors interact with peptide–zinc complexes is crucial for improving their bioavailability. However, this also presents an opportunity. By developing strategies to overcome these challenges, such as encapsulating peptide–zinc complexes or modifying the peptide structure to enhance stability, we can improve their bioavailability. Another opportunity lies in the development of new analytical and computational tools. These tools can help in accurately assessing peptide–zinc bioavailability and predicting the behavior of peptide–zinc complexes in the body, leading to more effective design of peptide–zinc-based products.

## Data Availability

Data will be made available on request.

## Supplementary Materials

The following supporting information can be downloaded at: https://www.mdpi.com/article/10.3390/biom15091311/s1, Table S1: Recently reported zinc chelating peptides from different marine protein sources.
