# Supplementary material for: Dual Perspectives on Peptide–Zinc Complexation: Highlighting Aquatic Sources While Contextualizing Other Natural Origins
Source: Biomolecules. 2025 Sep 12;15(9):1311. doi: 10.3390/biom15091311 (PMC12467604; doi:10.3390/biom15091311)
Supplement: Supplementary file 1 [file biomolecules-15-01311-s001.zip › biomolecules-3779758-supplementary.pdf]

Table 1

Recently reported zinc chelating peptides from different marine protein sources

Protein source	Protease	Select the type of zinc	Amino acid sequence (N to C terminal)	Molecular weight (Da)	Zinc chelating capacity	Antioxidant ability	Results of zinc absorption in vitro	Ref.
Tilapia scales	trypsin	zinc sulfate	ND	<1000 Da	13.2%	ND	ND	(Guo, Hong, & Yan, 2023)
	protease	zinc sulfate		1653Da	79.5%	ABTS-$^{+}$ radical scavenging activity assay：21.9 ± 0.9	Zinc solubility (pH 7.5)：27.98 ± 1.15％<br>Zinc released after peptic digestion：2.96 ± 0.72％<br>Zinc dialyzability：47.76 ± 1.32%	(Meng, Chen, Xia, & shen, 2021)
		zinc lactate			59.38%	ABTS-$^{+}$ radical scavenging activity assay：19.82 ± 0.36	Zinc solubility (pH 7.5)：52.07 ± 2.13%<br>Zinc released after peptic digestion：12.93 ± 1.14％<br>Zinc dialyzability：58.33 ± 1.63%	
	pepsin pancreatin	zinc lactate		1653 Da	33.25 ± 1.03%	ND	Zinc released after peptic digestion：10.29 ± 1.54％<br>Peptic-pancreatic digestion：44.05 ± 1.01％<br>Zinc dialyzability (bioaccessibility)：54.34 ± 2.55％	(L. Chen, Shen, & Xia, 2020)

				2745 Da	ND		Zinc released after peptic digestion：13.41 ± 0.92％<br>Peptic-pancreatic digestion：31.94 ± 0.27％<br>Zinc dialyzability (bioaccessibility)：45.35 ± 1.19％	
				4378 Da	19.44 ± 0.89%		Zinc released after peptic digestion：12.11 ± 0.9％<br>Peptic-pancreatic digestion：21.12 ± 0.19％<br>Zinc dialyzability (bioaccessibility)：33.23 ± 1.17％	
Oyster	pepsin	zinc sulfate	His-Leu-Arg-Gln-Glu-Glu-Lys-Glu-Glu-Val-Thr-Val-Gly-Ser-Leu-Lys	1882 Da	6.56 μg mg^{-1}	ND	ND	(D. Chen et al., 2013)
	alcalase		ND	ND	ND	Hydroxyl radical scavenging activity：45.79%		(Zhang, Zhou, Liu, & Zhao, 2018)
	alcalase				5.32 μg zinc/mg protein	ND		(Wang et al., 2021)

	pepsin		Glu-Val-Pro-Pro-Glu-Glu-His (EV PPEEH)	835.86 Da	Range: 26.54 to 101.08 mg zinc/g peptide			(Li et al., 2019)
Patinopecten yessoensis	alcalase	zinc sulfate	ND	ND	3mg mL^{-1}	ND	ND	(Liu et al., 2023)
Alaska pollock	pepsin	ND	Gly-Pro-Ala-Gly-Pro-His-Gly-Pro-Pro-Gly	844.4 Da	ND	ND	Increased zinc transport by 32.3%, decreased its retention and absorption by 60.2% and 13.8%, respectively.	(Q. Chen et al., 2017)
Antarctic krill	trypsin	zinc sulfate	ND	<1000 Da	115.70±2.25 mg/g	ND	Zinc solubility（time：90 min）：41.65±1.56%	(Sun et al., 2021)
Octopus	flavourzyme	zinc sulfate	ND	ND	52.06%	ND	ND	(Lin, Tang, Xu, & Wang, 2019)
Sea cucumbers	ND	zinc sulfate	Trp-Leu-Thr-Pro-Thr-Tyr-Pro-Glu	1005.5 Da	56.93%	ND	ND	(Liu et al., 2019)

			Ala-Ala-Tyr-Cys-Ala-Thr-Lys-Phe-Ala	945.1 Da	53.4%			
			Ala-Ala-Thr-Gly-Val-Met-Pro-Leu-Asp-Met	1005.2 Da	56.8%			
	alcalase		Trp-Leu-Thr-Pro-Thr-Tyr-Pro-Glu	1005.5 Da	33.31%			(X. Liu et al., 2019)
Silver carp	flavourzyme	$ZnCl_2$	Gly-Lys-Thr-Ala-Glu-Ile-Glu-Lys（GKKTAEIEK）	1003.15 Da	64.80 mg zinc/g peptide	ND	ND	(Jiang, Wang, Li, Wang, & Luo, 2014)
			Glu-Asp-Leu-Ala-Lys-Ala-Leu-Ala-Lys-Lys（EDLAKALAKK）	1086.28 Da	59.84 mg zinc/g peptide			
			Gln-Ala-Val-Glu-Ala-Gln-Lys（QAVEAQK）	772.85 Da	168.21 mg zinc/g peptide			

Lys-Glu-Leu-Glu-Glu-Lys （KELEEK）	774.86 Da	167.78 mg zinc/g peptide
Tyr-Glu-Glu-Ser-Gln-Ala-Glu-Leu-Glu-Gly-Ser-Leu-Lys （YEESQAELEGSLK）	1482.54 Da	131.53 mg zinc/g peptide
